# Intrauterine Infusion of Leukocyte-Poor Platelet-Rich Plasma Is an Effective Therapeutic Protocol for Patients with Recurrent Implantation Failure: A Retrospective Cohort Study

**DOI:** 10.3390/jcm12082823

**Published:** 2023-04-12

**Authors:** Yanna Ban, Xiaoliang Yang, Yan Xing, Wenjun Que, Zebo Yu, Wenwu Gui, Ying Chen, Xiru Liu

**Affiliations:** 1 Reproductive Medicine Center, The First Affiliated Hospital of Chongqing Medical University, No. 1 Youyi Road, Yuzhong District, Chongqing 400016, China; 2 Department of Blood Transfusion, The First Affiliated Hospital of Chongqing Medical University, No. 1 Youyi Road, Yuzhong District, Chongqing 400016, China

**Keywords:** recurrent implantation failure, leukocyte-poor platelet-rich plasma, live birth rate, embryo transfer

## Abstract

Background: The clinical application of autologous leukocyte-poor platelet-rich plasma (LP-PRP) in patients with recurrent implantation failure (RIF) is rare. This retrospective observational cohort study aimed to evaluate the efficacy of LP-PRP intrauterine infusion in patients with RIF. Methods: Patients with RIF undergoing frozen embryo transfer (FET) from January 2019 to December 2021 (*n* = 118) were enrolled, with those undergoing LP-PRP intrauterine infusion as the PRP group (*n* = 64), and those receiving no LP-PRP treatment as the control group (*n* = 54). The beta-human chorionic gonadotropin (β-hCG)-positive rate, clinical pregnancy rate (CPR), live birth rate (LBR), and miscarriage rate (MR) per ET cycle were compared. Results: The β-hCG-positive rate (57.8% vs. 38.9%, *p* = 0.041), CPR (45.3% vs. 24.5%, *p* = 0.022), and LBR per ET cycle (42.2% vs. 18.5%, *p* = 0.009) were higher in the PRP group than in the control group, and the three variables (62.5% vs. 41.2%, *p* = 0.040, 47.5% vs. 23.5%, *p* = 0.033, and 47.5% vs. 20.6%, *p* = 0.027) in the PRP group transferred with the *blastocyst-stage embryos* were also higher than those in the control group. The MR was similar in all groups. Conclusions: The LP-PRP treatment could improve the β-hCG-positive rate, CPR, and LBR in RIF patients undergoing FET cycles.

## 1. Introduction

Recurrent implantation failure (RIF) is a common issue, with an incidence of 10%, among patients undergoing assisted reproductive technology, despite developments in fertility treatment protocols [1,2]. Although there is no universal consensus on RIF definition, the commonly accepted definition is failure to achieve a clinical pregnancy after the transfer of at least four good-quality embryos during a minimum of three fresh or frozen cycles in a woman aged <40 years [3,4]. A number of factors, such as uterine, embryo, and immunologic status and substandard laboratory conditions, are associated with RIF [3]. After excluding embryo factors, poor endometrial receptivity is the main impediment to success for clinicians [5]. Many treatments have been developed to achieve successful implantation, including those using granulocyte colony-stimulating factor (G-CSF), human chorionic gonadotropin (HCG), immune modulators, growth factors, and endometrial scratch [6,7,8], but there is insufficient evidence to support the efficacy of these treatments [9].

Autologous platelet-rich plasma (PRP), including platelets (PLT), growth factors, cytokines, a certain amount of white blood cells (WBCs), and red blood cells (RBCs) depending on the preparation method, is derived from fresh peripheral blood with a platelet concentration above the baseline [10]. PRP is commonly applied in fields such as orthopedics, dermatology, and esthetic surgery [11,12,13]. The rationale behind these applications is that a series of growth factors, including platelet-derived growth factor (PDGF), vascular endothelial growth factor (VEGF), transforming growth factor-β (TGF-β), fibroblast growth factor (FGF), and epidermal growth factor (EGF), released from the α-granules in activated PRP, have regenerative, proliferative, angiogenic, chemotactic, proinflammatory, and antiapoptotic activity [14]. Intrauterine infusion of PRP was first reported in the treatment of patients with a thin endometrium [15]. These components have been identified to improve endometrial microvasculature and receptivity [16]. Recently, some studies have shown that the intrauterine infusion of PRP could increase the pregnancy rate in patients with RIF [17]. However, a few studies revealed no beneficial outcome, mainly due to the non-standardization of the PRP preparations and administration techniques [18].

Two PRP separation systems are usually used. Buffy coat-based systems produce PRP with a high concentration of leukocytes, defined as leukocyte-rich PRP (LR-PRP). In contrast, plasma-based systems with a low concentration of leukocytes are designed to separate only the platelet and plasma portions from whole blood, defined as leukocyte-poor PRP (LP-PRP) [19]. Previous studies have shown that LR-PRP intrauterine infusion can improve the pregnancy rate in patients with RIF. Despite LP-PRP’s potential to improve the formation of normal collagen and reduce the synthesis of inflammatory factors with fewer local adverse reactions than LR-PRP in patients with rotator cuff tears and knee osteoarthritis [20,21], the clinical application of LP-PRP is rare. To date, no meticulously designed studies regarding the effect of LP-PRP on endometrial receptivity have been published, and its beneficial effect requires further exploration [22]. The aim of this study was to determine the effect of autologous LP-PRP intrauterine infusion in patients with RIF and compare the outcomes with those of controls.

## 2. Materials and Methods

### 2.1. Population

A total of 118 patients with a history of RIF who underwent frozen embryo transfer (FET) at the Reproductive Medicine Center, The First Affiliated Hospital of Chongqing Medical University, China, from January 2019 to December 2021 were retrospectively analyzed. The inclusion criteria were (1) failure of clinical pregnancy after ≥3 ET cycles with at least 4 good-quality cleavage-/blastocyst-stage embryos; (2) women with RIF aged <40 years undergoing FET; (3) endometrium thickness ≥8 mm; (4) tubal factor infertility. The exclusion criteria were (1) availability of only poor-quality embryos; (2) congenital uterine abnormalities, untreated hydrosalpinges, endometriosis, adenomyosis, myoma, endometritis; (3) body mass index >30 kg/m^2^ or <18.5 kg/m^2^; (4) vitamin D deficiency; (5) uncontrolled endocrine, hematologic, or immunologic dysfunction; (6) endometrium thickness <8 mm; (7) severe male factor infertility; (8) thrombophilia and anticoagulant administration; (9) undergoing preimplantation genetic testing cycles or couples possessing genetic and chromosomal abnormalities. Finally, the patients receiving PRP treatment who provided a written informed consent were included in the PRP group (*n* = 64), and non-PRP patients were included in the control group (*n* = 54) according to patients’ personal willingness. All subjects gave their informed consent for inclusion before they participated in the study. This study was conducted in accordance with the Declaration of Helsinki, and the protocol was approved by the Ethics Committee of The First Affiliated Hospital of Chongqing Medical University (Approval No. 2022-0901).

All patients were treated using the antagonist protocol for controlled ovarian stimulation according to our routine hospital protocol. Basal hormonal, ultrasonography (USG), karyotype evaluations, and ovarian reserve and acquired thrombophilia testing were performed. If there was any suspected problem of the uterine cavity and endometrium detected by two-dimensional USG, three-dimensional USG and confirmatory hysteroscopy were performed. The male partners were also evaluated and treated if necessary.

Basic patient information, basal hormonal evaluation results, serum beta hCG (β-hCG)-positive rate per ET cycle, clinical pregnancy rate (CPR), live birth rate (LBR), and miscarriage rate (MR) were determined. The primary outcome measures were CPR and LBR, defined as ultrasonographic confirmation of a live intrauterine pregnancy and after at least 24 weeks of pregnancy per transfer cycle, respectively. The secondary outcomes were β-hCG-positive rate per ET cycle and MR, defined as a serum β-hCG level > 5 mIU/mL and termination of pregnancy with an ultrasound confirmation before 20 weeks, respectively.

### 2.2. PRP Preparation

First, we selected the appropriate peripheral vein for puncture. PRP was prepared using the NIGALE Blood Composition Separator (NGLXCF-3000), manufactured by NIGALE Sichuan Biomedical Co., Ltd. in Chengdu City, Sichuan Province, China. In brief, self-inspection was completed after the startup process, the PLT separation program was selected, and the parameters were set. The consumables were installed according to the instructions, and the sodium citrate anticoagulant was added automatically by a machine at an anticoagulant/blood proportion of 1:11. After initiating the blood collection preparation, venipuncture was performed with a 16G needle after disinfection. The “blood collection” button was pressed to run the machine automatically. Whole blood (1 mL) was left aside to obtain the PLT and white blood cell (WBC) count. Then, about 300 mL of whole blood was drawn into a machine for centrifugation, and the PLT was resuspended with plasma to obtain the autologous PRP (approximately 20 mL). Finally, the remaining RBC and plasma left in the machine were automatically transfused back into the patient. The whole collection process was finished after one cycle of separation.

The collected PRP was divided into eight bags (approximately 2.5 mL/bag) using sterile tubing welders (XL-100, Shanghai Lailing Biomedical Co., Ltd. in Shanghai City, China) and a heat machine (GIR-III, Suzhou Medical Equipment Factory Co., Ltd. Suzhou City, Jiangsu Province, China). The fresh PRP from one bag was infused immediately, and the remaining seven bags (Q-100, NIGALE Sichuan Biomedical Co., Ltd. in Chengdu City, Sichuan Province, China) were kept in a refrigerator (DW-HL398, Zhongke Meiling Cryogenic Technology Co., Ltd. in Hefei City, Anhui Province, China) at –80 °C [23]. For patients in the PRP group, the baseline PLT count before PRP collection, the PLT and WBC count in PRP, and the PLT enrichment coefficient (PEC = PLT count in PRP/PLT count at baseline) were determined. The fresh PRP was administered within 2 days of preparation.

### 2.3. FET Protocol

The endometrium was prepared by a hormone replacement treatment protocol. After ovarian quiescence was confirmed by vaginal ultrasound, the endometrial preparation for ET was performed on days 2–3 of menstruation with increasing estradiol valerate (Progynova, Bayer, Germany) or femoston (Abbott Healthcare Products, USA) administration (4 mg/d for 4 days orally, 6 mg/d for 4 days, and 6 mg/d or 8 mg/d for the last 4–8 days according to the endometrial thickness). After 12–16 days on estrogens, the endometrial thickness (EMT) and triple-layer pattern were measured using vaginal ultrasound. When EMT ≥ 8 mm was achieved, progesterone supplementation was initiated vaginally (progesterone vaginal sustained-release gel, 90 mg once daily, Crinone, Merck, Switzerland) and oral dydrogesterone administration (20 mg twice daily, Duffetone, Abbott Healthcare Products, USA). Estradiol (E2) and progesterone (P) were determined to ensure that no spontaneous ovulation had occurred. The method of progesterone supplementation was based on the hospital protocol for luteal phase support.

On the day before ET, endometrial thickness was determined using vaginal ultrasound, and the estradiol (E2) and progesterone (P) concentrations were measured. If serum P ≥ 10 ng/mL and E2 ≥ 100 pg/mL, ET was scheduled. The ET was performed on day 4 of P supplementation for day 3 cleavage-stage embryos and on day 6 of P supplementation for blastocyst-stage embryos. Then, 2 good-quality cleavage-stage embryos with at least 1 grade I embryo determined according to the Istanbul consensus [24] or 1–2 good-quality blastocyst-stage embryos (grade A or B) graded according to Gardner’s classification [25] were transferred on the day of thawing. The clinicians routinely suggested choosing the transfer of double embryos in the following FET after failure in the cycle of fresh ET. However, one single-embryo transfer could be executed in the situation of one embryo left following ET, scarred uterus, and as a precaution against the risk of multiple pregnancies. If pregnancy occurred, luteal phase support was continued for 10–11 weeks of gestation.

### 2.4. PRP Administration Technique

Approximately 1 mL of autologous LP-PRP in the syringe connected to the ET catheter was infused into the uterine cavity using vaginal ultrasound guidance every time. After autologous PRP intrauterine infusion, the patient was left in bed for 10–15 min. Adverse events (bleeding, pain, emesis, etc.) were observed within 4 h of PRP intrauterine infusion. Two autologous LP-PRP intrauterine infusions per FET cycle were performed. One bag of fresh LP-PRP could be used to perform intrauterine infusion directly on day 1 of P supplementation. The other bag of frozen LP-PRP was activated by sudden heat shock in the Department of Blood Transfusion before intrauterine infusion: frozen LP-PRP stored at −80 °C was immersed in liquid nitrogen for 5 min and quickly warmed at 37 °C for 5 min, twice [22,26]. The activated frozen LP-PRP was administered two days before ET.

### 2.5. Statistical Analysis

Statistical analysis was performed using SPSS version 22.0 (IBM Corp, Armonk, NY, USA). The continuous variables are presented as means ± standard deviations for normally distributed data, and Student’s *t*-test was used to compare the differences in patient characteristics. Multiple comparison analysis was performed using post hoc ANOVA. Non-normal-distribution variables are presented as medians (interquartile ranges, IQRs), and the differences between groups were tested using the Mann–Whitney U test. The categorical variables are presented as frequencies (percentages) and were compared using the chi-square test. Logistic regression analysis was performed to evaluate the effect of the variables on CPR. Statistical significance was set at *p* < 0.05.

## 3. Results

### 3.1. Patient Characteristics

**Figure 1:**
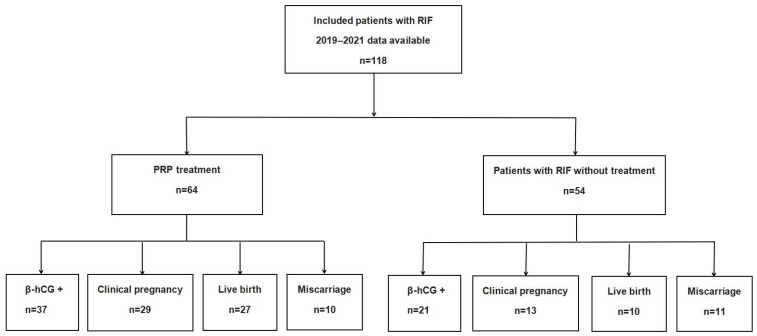
Flow chart of the study population.

Figure 1 depicts the flowchart of the study population. The baseline characteristics of all patients are summarized in Table 1. A total of 118 patients were divided into 2 groups according to the presence or absence of PRP intrauterine infusion: 64 patients in the PRP group and 54 in the control group. The baseline characteristics included age, body mass index (BMI), anti-Mullerian hormone, follicle-stimulating hormone (FSH), luteinizing hormone (LH), estradiol (E2), and progesterone (P) levels, and the duration of infertility, number of previous ET attempts, endometrial thickness before and after two PRP infusions, and diagnosis of infertility were similar in both groups (Table 1, Table 2 and Table 3). In addition, the numbers of oocytes retrieved, available embryos, and good-quality embryos for blastocyst- or cleavage-stage ET between the PRP and control groups showed no differences (Table 2 and Table 3).

### 3.2. Outcomes

The pregnancy outcome variables between the PRP and the control groups are listed in Table 4. The β-hCG-positive rate (57.8%, 37/64 vs. 38.9%, 21/54), CPR per ET cycle (45.3%, 29/64 vs. 24.1%, 13/54), and LBR per ET cycle (42.2%, 27/64 vs. 18.5%, 10/54; *p* = 0.009) were significantly higher in the PRP group than in the control group (*p* < 0.05), respectively. Although the MR appeared to be lower in the PRP group than in the control group, the difference between the two groups was not significant (15.6%, 10/64 vs. 20.4%, 11/54; *p* > 0.05). No serious adverse events (bleeding, pain, emesis, infection) were found in the PRP group, as shown in Table S1.

Patients with blastocyst-stage ET in the PRP group (*n* = 40) experienced better outcomes than those in the control group (*n* = 34). The β-hCG-positive rate (65.0%, 26/40 vs. 41.2%, 14/34, *p* = 0.04), CPR per ET cycle (47.5%, 19/40 vs. 23.5%, 8/34, *p* = 0.03), and LBR per ET cycle (47.5%, 19/40 vs. 20.6%, 3/20; *p* = 0.027) in these patients were higher in the PRP group than in the control group. The MR was similar in the two groups (*p* > 0.05) (Table 2). Furthermore, the proportion of good blastocysts in the PRP group was less than in the control group (*p* = 0.03). A statistically significant number of patients with PRP treatment had higher odds of pregnancy than those in the control group.

**Figure 2:**
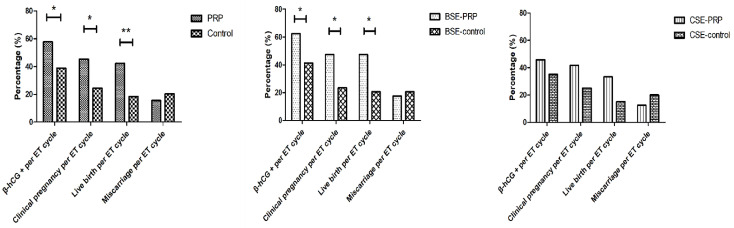
Comparison of the outcome variables in different groups, including the β-hCG-positive rates, clinical pregnancy rate, live birth rate, and miscarriage rate per ET cycle in the PRP (*n* = 64), control (*n* = 54), blastocyst-stage-embryo (BSE) (*n* = 40), BSE control (*n* = 34), cleavage-stage-embryo (CSE) (*n* = 24), and CSE control (*n* = 20) groups.* Statistically significant: *p* < 0.05, ** Statistically significant: *p* < 0.01.

For those patients who had cleavage-stage ET in the PRP group (*n* = 24), the proportion of top-quality embryos was not significantly different from that in the control group (*p* = 0.09). Although the PRP group showed higher β-hCG-positive rate (45.8%, 11/24 vs. 35.0%, 7/20, *p* = 0.226), CPR per ET cycle (41.7%, 10/24 vs. 25.0%, 5/20, *p* = 0.246), and LBR per ET cycle (33.3%, 8/24 vs. 15.0%, 3/20, *p* = 0.294), no significant differences were observed between the two groups. Moreover, the MR was also similar in the two groups (*p* = 0.498) (Table 3). In addition, the outcome variables of all groups, including the β-hCG-positive rate, CPR per ET cycle, LBR per ET cycle, and MR, are displayed in Figure 2.

Table 5 shows the baseline and the comparisons of PRP quality variables in β-hCG-positive (the concentrations of PLT and WBC were 745.56 *±* 189.59 × 10^9^/L and 553.33 *±* 228.98 × 10^6^/L), clinical pregnancy (the concentrations of PLT and WBC were 758.44 ± 428.81 × 10^9^/L and 550.74 ± 286.36 × 10^6^/L), and non-pregnancy groups (the concentrations of PLT and WBC were 652.67 ± 140.31 × 10^9^/L and 496.33 ± 179.42 × 10^6^/L) who accepted PRP intrauterine infusion. No significant between-group differences were identified in the PLT count at baseline, PLT count in PRP, PEC, and WBC count in PRP, indicating that the PRP preparation was standardized in all groups.

The results of the univariate logistic regression analysis regarding the associations between PRP treatment, age, body mass index, anti-Mullerian hormone, LH, estradiol (E2) and progesterone (P) levels, duration of infertility, number of previous ET attempts, and endometrial thickness and the CPR outcome during the FET cycle are presented in Table 6. The PRP treatment and FSH level were positive factors associated with the outcome of CPR (*p* = 0.018 and *p* = 0.011). However, the multivariate regression analysis showed that only the PRP treatment (odds ratio 2.750, 95% confidence interval 1.136–6.660) had independent effects on the outcome of CPR (Table 6). Moreover, a sub-analysis according to the number of blastocyst-stage embryos transferred was performed. Similar results are also presented in Table S2.

## 4. Discussion

RIF is a challenging problem in the field of assisted reproductive technology. Recently, abnormal endometrial receptivity is considered a risk factor for embryo implantation failures [27]. The results of this study indicated that PRP intrauterine infusion appeared to improve *β-hCG-positive rate*, CPR, and LBR during FET cycles in women with RIF, which is in agreement with other research [7]. This is the first study reporting the efficacy of LP-PRP intrauterine infusion in patients with RIF. However, the data showed no advantage of using the LP-PRP treatment for MR improvement. Recently, Li M. et al. reported that the PRP group had better outcomes in terms of clinical pregnancy, live birth, implantation, and positive *β-hCG* 14 days after embryo transfer and showed no advantages in improving the miscarriage rate, which is consistent with our results [28].

According to the results of the blastocyst-stage embryos (BSE)/cleavage-stage embryos (CSEs) transfer, further analysis of the data revealed that statistical differences in the *β-hCG-positive rate*, CPR, and LBR were only found between the BSE, PRP, and BSE control groups, which is similar to observations in other studies [29,30], whereas the three variables were similar in the CSE, PRP, and CSE control groups. This is a different result from that obtained for women with a history of RIF, whereby the PRP treatment appeared to improve the FET outcomes, with an increase in CPR and LBR [22]. In fact, there was a clear trend in this dataset (Table 3), but the small sample size was insufficient to show statistically significant results. As such, studies with large sample sizes are needed to verify this conclusion. The same is true for the BSE groups, which should have been subdivided into one-embryo (PRP *n* = 16, control *n* = 8) and two-embryo (PRP *n* = 25, control *n* = 26) transfer groups. Although the sample size was too small, the advantages of the PRP treatment displayed a notable positive trend. Moreover, there is the possibility that some of the PRP was flushed out after intrauterine infusion, and the actual quantity retained in the cavity could be less than that administered. However, the injection of PRP into the sub-endometrium did not provide any more advantages than PRP intrauterine infusion [22]. Therefore, this effect could be ignored due to our standardized intrauterine infusion techniques.

To our knowledge, research on the efficacy of PRP intrauterine infusion for patients with RIF is ongoing. Positive results have been reported in several studies [29,30,31]. However, a few studies did not show a significant improvement with the PRP treatment [18], which could be related to differences in PRP preparation and administration techniques. According to the different preparation methods, PRP can be divided into LR-PRP derived from buffy coat and LP-PRP [19]. The WBC count (533 × 10^6^/L) in our PRP sample, defined as LP-PRP, was lower than that in LR-PRP (7991 × 10^6^/L) [32]. LP-PRP is an unusual treatment utilized in major RIF studies, and the effect of LP-PRP on intrauterine infusion was rarely evaluated. Our study revealed that LP-PRP intrauterine infusion could improve FET outcomes in RIF patients, with an increase in the β-hCG-positive rate, CPR, and LBR.

In this study, some advantages of PRP preparation using an automatic Blood Composition Separator were noted compared to the manual preparation mentioned in the literature. First, according to the equipment operation protocol, the entire process was conducted in a completely closed state, which could avoid potential contamination caused by the manual preparation. No infections were found in the PRP group. Second, the simple process only required 15–20 min, which was less than the time required by the manual methods (~45 min) [33]. In this way, the product quality can be effectively controlled with less dependence on the proficiency of different operators. Third, the PLT count in PRP could generally reach 3–5 times the baseline PLT count owing to the most significant effect on tissue repair of PRP, with enrichment in the range of 2–6-fold [34]. Moreover, quite low RBC and WBC mixed counts indicated that the PRP in this study was safe and of high purity. Fourth, the PRP could be divided into eight bags for multiple uses by a single collection in this study. This would be advantageous to reduce the costs. Finally, in our study, RBC, WBC, and plasma, except PRP, were transfused back into patients after separation to avoid blood wastage and hypovolemia, unlike what is done when using the manual methods. Therefore, PRP preparation using an automatic Blood Composition Separator has been recommended as the preferred method in China [23].

As we know, the debate on the criteria for PRP intrauterine infusion schedule for a patient with RIF is ongoing. The time of PRP infusion differs between studies [35]. Therefore, the best time interval between PRP infusion and ET needs to be verified through a series of research studies. The schedule in our study was two PRP infusions on day 1 of P supplementation and two days before ET. One PRP intrauterine infusion on day 1 of P supplementation could improve the outcome of RIF patients, with an increase in the ongoing pregnancy rate and live birth rate [22], whose schedule was in accordance with our first PRP infusion. Moreover, a PRP infusion before day 2 ET was a major choice with a positive effect on the pregnancy outcomes of patients with RIF [36], and this timing is the same as that of our second PRP infusion. Due to the limitation of the growth in endometrial thickness after progesterone administration, the mechanism behind this was hypothesized as depending on PRP, which includes various growth factors and cytokines, possibly improving endometrial receptivity and consequent implantation [18]. Since no patients with a thin endometrium were enrolled in this study, we combined two PRP infusions on the endometrial transformation day and two days before implantation, respectively, to further enhance endometrial receptivity.

Since progesterone limits the possibility of real endometrial growth, the potential biological effect of PRP infusion could be explained by proinflammatory, chemotactic, angiogenic, and antiapoptotic effects [18]. Among them, the Th1/Th2 imbalance of the intrauterine environment was a major reason for RIF in patients [37]. A low expression of TNF-α mediated by Th1 may reduce the expression of adhesion molecules and block endometrial angiogenesis, which causes embryo implantation failure; additionally, TNF-α over-expression could inhibit endometrium decidualization, shorten the survival time of epithelial cells, and ultimately promote the apoptosis of endometrial cells [38]. IL-6 secreted by Th2 plays an important role in embryo implantation, including endometrium decidualization, angiogenesis, tissue remodeling, and trophoblast differentiation [39]. Therefore, it is possible that fresh LP-PRP in this study, including an appropriate amount of WBC, unlike LR-PRP, regulated the Th1/Th2 balance in the patients and induced the local immune balance to tilt toward Th2 to improve the intrauterine proinflammatory environment, which is conducive to embryo implantation and angiogenesis [37]. The other frozen PRP activated by sudden heat shock, which releases more growth factors when alpha granules in the platelets break down, could improve endometrial receptivity in the following FET cycle [22,26]. The unclear mechanism of action of PRP remains to be explored in future research.

The strength of our study is that we utilized LP-PRP intrauterine infusion prepared by a machine for the first time to improve the *β-hCG-positive rate*, CPR, and LBR during FET cycles in patients with RIF rather than common LR-PRP. Second, both the fresh PRP and the activated frozen PRP were utilized per FET cycle, which is different from the majority of protocols. Finally, the PRP preparation technique and storage condition in this study were standardized [23].

However, there are certain limitations in this study. First, the efficacy of LP-PRP and LR-PRP was not compared simultaneously in this study because no LR-PRP preparation is carried out in our center, despite a series of reports revealing the efficacy of LR-PRP in RIF. Second, this is a review of data from a single center, which might have led to inherent biases and requires further external validation. Prospective randomized controlled studies with large cohorts in the LR-PRP and LP-PRP groups are required due to the limited sample size and lack of LR-PRP preparation. Finally, the heterogeneity of the preparations and the mechanism of LP-PRP in improving CPR and LBR are unclear, so further research is necessary to clarify these issues.

## 5. Conclusions

The LP-PRP treatment can improve the *β-hCG-positive rate*, CPR, and LBR during FET cycles in women with RIF. However, the data of our study did not reveal any advantage of the PRP treatment for MR. While the data in this study indicated that the LP-PRP treatment has the potential to improve the live birth rate for women with a history of RIF, a prospective large randomized controlled trial is required to generate high-quality evidence on its clinical use in patients with RIF.

## Figures and Tables

**Table 1:** Baseline characteristics of the PRP and non-PRP groups.

Variables	PRP Group (*n* = 64)	Non-PRP Treatment Group (*n* = 54)	*p*-Value
Age (years)	32.04 ± 4.36	32.22 ± 4.31	0.916
BMI (kg/m^2^)	20.69 ± 4.36	22.52 ± 2.99	0.713
AMH (ng/mL)	4.61 (2.25, 7.31)	4.49 (2.64, 7.32)	0.884
Basal FSH (U/L)	6.64 ± 3.21	5.78 ± 2.27	0.236
Basal LH (mIU/mL)	5.19 (3.34, 10.43)	5.12 (3.39, 6.93)	0.223
Basal E2 (pg/mL)	75.26 (44.25, 124.5)	67.06 (44.92, 103.8)	0.461
Basal progesterone (ng/mL)	0.59 (0.36, 0.91)	0.53 (0.36, 0.76)	0.746
Diagnosis of infertility			
Tubal factors	41 (64.1%)	37 (68.5%)	0.697
Polycystic ovary syndrome	18 (28.1%)	14 (25.9%)	0.838
Unexplained infertility	5 (7.8%)	3 (5.6%)	0.725
Duration of infertility (years)	3 (2,4)	3.5 (2,5)	0.310
No. of the blastocyst-stage embryos transferred	40 (62.5%)	34 (63.0%)	1.000
No. of the cleavage-stage embryos transferred	24 (37.5%)	20 (37.0%)	1.000
No. of previous ET attempts	3 (3, 4)	3 (3, 4)	0.826
Endometrial thickness before first PRP infusion (mm)	10.88 ± 1.83	10.95 ± 1.66	0.795
Endometrial thickness on the ET day after two PRP infusions (mm)	11.64 ± 2.06	11.29 ± 1.76	0.366

BMI: body mass index, AMH: anti-Mullerian hormone, FSH: follicle-stimulating hormone, LH: luteinizing hormone, E2: estradiol, ET: embryo transfer, PRP: platelet-rich plasma.

**Table 2:** Baseline characteristics and outcome variables of the blastocyst-stage-embryo group.

Variables	PRP Group (*n* = 40)	Non-PRP Treatment Group (*n* = 34)	*p*-Value
Age (years)	31.53 ± 4.03	32.0 ± 4.20	0.806
BMI (kg/m^2^)	23.23 ± 3.25	22.76 ± 3.12	0.398
AMH (ng/mL)	5.07 (2.58, 7.39)	3.76 (2.87, 6.74)	0.488
Basal FSH (U/L)	6.06 ± 2.24	5.44 ± 2.21	0.392
Basal LH (mIU/mL)	5 (3.34, 10.12)	4.64 (3.49, 6.59)	0.368
Basal E2 (pg/mL)	93.33 (47.95, 149.25)	68 (43.84, 109.65)	0.300
Basal progesterone (ng/mL)	0.57 ± 0.24	0.52 ± 0.22	0.428
Duration of infertility (years)	3 (2, 4)	3 (2, 5)	0.332
No. of previous ET attempts	3 (3, 4)	3 (3, 4)	0.825
No. of oocytes retrieved	15.18 ± 1.25	14.18 ± 0.93	0.276
No. of available embryos	8.32 ± 0.72	9.21 ± 0.67	0.135
No. of good-quality embryos	5.68 ± 0.38	5.59 ± 0.32	0.491
No. of blastocysts transferred			0.218
1	15 (37.5%)	8 (23.5%)	
2	25 (62.5%)	26 (76.5%)	
Endometrial thickness before first PRP infusion (mm)	10.84 ± 2.00	11.34 ± 1.59	0.159
Endometrial thickness on the ET day after two PRP infusions (mm)	11.72 ± 2.09	11.63 ± 1.62	0.905
No. of good-quality BSE (grade A or B) transferred per ET cycle	1 (1, 1)	2 (1, 2)	0.030 *
β-hCG+ per ET cycle	26 (65.0%)	14 (41.2%)	0.040 *
Clinical pregnancy per ET cycle	19 (47.5%)	8 (23.5%)	0.033 *
Live birth per ET cycle	19 (47.5%)	7 (20.6%)	0.027 *
Miscarriage per ET cycle	7 (17.5%)	7 (20.6%)	0.773

BMI: body mass index, AMH: anti-Mullerian hormone, FSH: follicle-stimulating hormone, LH: luteinizing hormone, E2: estradiol, β-hCG+: beta hCG-positive, ET: embryo transfer, PRP: platelet-rich plasma. * Statistically significant: *p* < 0.05.

**Table 3:** Baseline characteristics and outcome variables of the cleavage-stage-embryo group.

Variables	PRP Group (*n* = 24)	Non-PRP Treatment Group (*n* = 20)	*p*-Value
Age (years)	32.90 ± 4.86	32.60 ± 4.57	0.822
BMI (kg/m^2^)	21.42 ± 2.85	22.11 ± 2.78	0.706
AMH (ng/mL)	4.90 ± 3.72	5.68 ± 3.32	0.234
Basal FSH (U/L)	7.81 ± 4.15	6.36 ± 2.31	0.340
Basal LH (mIU/mL)	7.51 ± 4.31	5.43 ± 2.41	0.248
Basal E2 (pg/mL)	67.63 ± 52.17	72.28 ± 38.45	0.777
Basal progesterone (ng/mL)	0.58 ± 0.32	0.56 ± 0.24	0.925
Duration of infertility (years)	4.53 ± 2.23	4.15 ± 2.21	0.693
No. of previous ET attempts	3.5 (3, 4)	3.5 (3, 4.75)	0.969
Endometrial thickness before first PRP infusion (mm)	11.02 ± 1.56	10.29 ± 1.61	0.147
Endometrial thickness on the ET day after two PRP infusions (mm)	11.51 ± 2.04	10.71 ± 1.88	0.167
No. of oocytes retrieved	14.40 ± 2.21	11.95 ± 1.16	0.759
No. of available embryos	6.75 ± 0.82	7.70 ± 0.69	0.234
No. of good-quality embryos	3.60 ± 0.15	3.45 ± 0.13	0.433
No. of grade I embryos transferred per ET cycle	0 (0, 1)	0 (0, 1.75)	0.947
No. of grade II embryos transferred per ET cycle	1 (0.25, 2)	1 (0, 2)	0.940
β-hCG + per ET cycle	11 (45.8%)	7 (35.0%)	0.226
Clinical pregnancy per ET cycle	10 (41.7%)	5 (25.0%)	0.246
Live birth per ET cycle	8 (33.3%)	3 (15.0%)	0.294
Miscarriage per ET cycle	3 (12.5%)	4 (20.0%)	0.498

BMI: body mass index, AMH: anti-Mullerian hormone, FSH: follicle-stimulating hormone, LH: luteinizing hormone, E2: estradiol, β-hCG+: beta hCG-positive, ET: embryo transfer, PRP: platelet-rich plasma.

**Table 4:** Comparison of outcome variables.

Variables	PRP Group (*n* = 64)	Non-PRP Treatment Group (*n* = 54)	*p*-Value
β-hCG+ per ET cycle	37 (57.8%)	21(38.9%)	0.041 *
Clinical pregnancy per ET cycle	29 (45.3%)	13 (24.1%)	0.022 *
Live birth per ET cycle	27 (42.2%)	10 (18.5%)	0.009 **
Miscarriage per ET cycle	10 (15.6%)	11 (20.4%)	0.630

β-hCG+: beta hCG-positive, ET: embryo transfer, PRP: platelet-rich plasma. * Statistically significant: *p* < 0.05, ** Statistically significant: *p* < 0.01.

**Table 5:** Multiple comparisons to evaluate statistical differences in the PRP group.

Variables	PRP Group (*n* = 64)			*p*-Value	
	β-hCG+ (*n* = 37)	CP (*n* = 29)	Non-Pregnancy (*n* = 27)	β-hCG+ vs. CP	β-hCG+ vs. Non-Pregnancy
Baseline platelet count (10^9^/L)	240.48 ± 51.50	231.07 ± 47.51	217.07 ± 37.72	0.978	0.170
Platelet count in PRP (10^9^/L)	745.56 ± 189.59	758.44 ± 428.81	652.67 ± 140.31	0.894	0.355
Platelet enrichment coefficient (PEC)	3.05 ± 0.72	3.27 ± 1.37	3.03 ± 0.51	0.701	0.849
WBC count in PRP (10^6^/L)	553.33 ± 228.98	550.74 ± 286.36	496.33 ± 179.42	0.917	0.770

WBC: white blood cell, β-hCG+: beta hCG-positive, CP: clinical pregnancy, PRP: platelet-rich plasma.

**Table 6:** Logistic regression analysis to evaluate the effect of variables, including platelet-rich plasma treatment, on the clinical pregnancy rate.

**Univariate Analysis**			
**Variables**	**Odds Ratio**	**95% CI**	***p*-Value**
Age (years)	1.038	(0.949–1.135)	0.419
BMI (kg/m^2^)	0.963	(0.850–1.092)	0.556
AMH (ng/mL)	0.921	(0.821–1.033)	0.159
Basal FSH (U/L)	1.230	(1.049–1.442)	0.011 *
Basal LH (mIU/mL)	0.971	(0.883–1.068)	0.543
Basal E2 (pg/mL)	0.997	(0.990–1.003)	0.325
Basal progesterone (ng/mL)	0.788	(0.396–1.571)	0.499
Duration of infertility (years)	0.873	(0.717–1.064)	0.178
Previous ET attempts	1.025	(0.730–1.440)	0.887
Endometrial thickness during FET cycle (mm)	0.809	(0.641–1.020)	0.073
PRP group	2.613	(1.181–5.785)	0.018 *
**Multivariate Analysis**			
**Variables**	**Odds Ratio**	**95% CI**	***p*-Value**
Age (years)	0.997	(0.895–1.111)	0.960
BMI (kg/m^2^)	0.958	(0.831–1.104)	0.552
AMH (ng/mL)	0.936	(0.830–1.057)	0.287
Basal FSH (U/L)	1.182	(0.984–1.420)	0.075
Basal LH (mIU/mL)	0.947	(0.843–1.064)	0.361
Basal E2 (pg/mL)	0.999	(0.991–1.007)	0.743
Basal progesterone (ng/mL)	0.859	(0.389–1.897)	0.706
Duration of infertility (years)	0.864	(0.696–1.071)	0.183
Previous ET attempts	1.050	(0.705–1.565)	0.810
Endometrial thickness during FET cycle (mm)	0.825	(0.640–1.063)	0.137
PRP group	2.750	(1.136–6.660)	0.025 *

BMI: body mass index, AMH: anti-Mullerian hormone, FSH: follicle-stimulating hormone, LH: luteinizing hormone, E2: estradiol, ET: embryo transfer, FET: frozen embryo transfer, β-hCG+: beta hCG-positive, PRP: platelet-rich plasma. * Statistically significant at *p* < 0.05.

## Data Availability

The original contributions presented in the study are included in the article/Supplementary Material. Further inquiries can be directed to the corresponding author.

## Supplementary Materials

The following supporting information can be downloaded at: https://www.mdpi.com/article/10.3390/jcm12082823/s1, Table S1: Side-effects in PRP group; Table S2: Logistic regression analysis to evaluate the effect of variables including platelet-rich plasma treatment on clinical pregnancy rate in blastocyst-stage embryo group.
