# Supplementary material for: Intrauterine Infusion of Leukocyte-Poor Platelet-Rich Plasma Is an Effective Therapeutic Protocol for Patients with Recurrent Implantation Failure: A Retrospective Cohort Study
Source: J Clin Med. 2023 Apr 12;12(8):2823. doi: 10.3390/jcm12082823 (PMC10146382; doi:10.3390/jcm12082823)
Supplement: Supplementary file 1 [file jcm-12-02823-s001.zip › jcm-2191035-supplementary.pdf]

Table S1. Side-effects in PRP group.

	**PRP group（n=64）**
Bleeding	0 (0%)
Pain	0 (0%)
Emesis	0 (0%)
Infection	0 (0%)

Table S2. Logistic regression analysis to evaluate the effect of variables including platelet-rich plasma treatment on clinical pregnancy rate in blastocyst-stage embryo group.

**Univariate analysis Variables**	**Odds ratio**	**95% CI**	***P*-value**
Age (years)	0.947	(0.834-1.060)	0.312
BMI (kg/m2)	1.015	(0.872-1.181)	0.849
AMH (ng/mL)	1.104	(0.954-1.279)	0.184
Basal FSH(U/L)	0.764	(0.601-0.970)	0.027*
Basal LH (mIU/mL)	1.038	(0.916-1.177)	0.557
Basal E2 (pg/mL)	1.004	(0.996-1.012)	0.326
Basal progesterone (ng/mL)	0.815	(0.310-1.382)	0.849
Duration of infertility (years)	1.044	(0.812-1.344)	0.736
Previous ET attempts	0.968	(0.644-1.455)	0.877
No. of blastocysts transferred	0.898	(0.321-2.513)	0.838
Endometrial thickness during FET cycle (mm)	0.908	(0.712-1.153)	0.116
PRP group	2.940	(1.075-8.045)	0.036*
**Multivariate analysis Variables**	**Odds ratio**	**95% CI**	***P*-value**
Age (years)	0.947	(0.811-1.107)	0.496
BMI (kg/m^2)	1.008	(0.835-1.218)	0.933
AMH (ng/mL)	1.098	(0.932-1.293)	0.264
Basal FSH (U/L)	0.800	(0.578-1.106)	0.177
Basal LH (mIU/mL)	1.061	(0.908-1.239)	0.456
Basal E2 (pg/mL)	1.044	(0.993-1.015)	0.458
Basal progesterone (ng/mL)	0.918	(0.107-1.732)	0.974
Duration of infertility (years)	1.025	(0.765-1.374)	0.867
Previous ET attempts	0.979	(0.564-1.701)	0.941
No. of blastocysts transferred	1.076	(0.284-4.078)	0.915
Endometrial thickness during FET cycle (mm)	0.978	(0.764-1.172)	0.228
PRP group	4.141	(1.014-16.917)	0.048*

BMI: body mass index, AMH: antimullerian hormone, FSH: follicle-stimulating hormone, LH: luteinizing hormone, E2: estradiol, ET: embryo transfer, FET: frozen embryo transfer, β-hCG+: beta hCG positive, PRP: platelet-rich plasma. *Statistically significant at $P<0.05$.
